# Protein engineering strategies for rational immunogen design

**DOI:** 10.1038/s41541-021-00417-1

**Published:** 2021-12-17

**Authors:** Timothy M. Caradonna, Aaron G. Schmidt

**Affiliations:** 1 Ragon Institute of MGH, MIT and Harvard, Cambridge, MA 02139 USA; 2 Department of Microbiology, Harvard Medical School, Boston, MA 02115 USA

**Keywords:** Antibodies, Virology, Protein vaccines

## Abstract

Antibody immunodominance refers to the preferential and asymmetric elicitation of antibodies against specific epitopes on a complex protein antigen. Traditional vaccination approaches for rapidly evolving pathogens have had limited success in part because of this phenomenon, as elicited antibodies preferentially target highly variable regions of antigens, and thus do not confer long lasting protection. While antibodies targeting functionally conserved epitopes have the potential to be broadly protective, they often make up a minority of the overall repertoire. Here, we discuss recent protein engineering strategies used to favorably alter patterns of immunodominance, and selectively focus antibody responses toward broadly protective epitopes in the pursuit of next-generation vaccines for rapidly evolving pathogens.

## B cell immunodominance

Antibody (Ab) responses raised against complex protein antigens can preferentially target particular epitopes in a reproducible hierarchy, a phenomenon known as immunodominance. These primary targets of Ab responses are often immunologically dominant, while those engaged by minor portions of the overall response are considered immunologically subdominant. This asymmetry contributes to the host-pathogen ‘arms race’, where particular regions of surface-exposed antigens experience the most immune pressure, and are subsequently key sites of antigenic variation^1,2^. Viral antigens like influenza hemagglutinin (HA) or HIV envelope protein (Env), have conserved structural or functional regions. Abs targeting these epitopes are often broadly neutralizing (bnAbs) or protective (bpAbs), the latter through Fc-mediated effector functions^3^. However, such antibodies are generally immunologically subdominant, and make up a minority of the overall repertoire. Next-generation vaccines for rapidly evolving pathogens aim to alter patterns of dominance to elicit higher levels of broadly neutralizing or protective responses.

While B cell immunodominance is an incompletely understood phenomenon, there are several key aspects influencing inter-clonal competition in the germinal center (GC) reaction that can be leveraged for rational immunogen design^4^. (1) Precursor frequency, the number of naïve B cells that engage a specific epitope, is a key limiting factor; if fewer B cells engage an epitope, the greater the likelihood that the subdominant-directed population will be outcompeted by more abundant B cell clones. This is a limiting factor for many bnAb precursors such as VRC01-class Abs targeting the HIV Env CD4 binding site (CD4bs), which are present at very low frequencies in human repertoires^5^. Precursor frequency may also be influenced by central tolerance as is the case for certain autoreactive HA-stem antibodies; negative selection attempts to remove these autoreactive B cells from the naïve repertoire^6,7^. The accessibility of a given epitope can also contribute, as an epitope must be accessible to BCRs in order to trigger an antibody response. (2) Precursor affinity for the antigen drives GC establishment or entry, as high affinity for antigen is linked with increased acquisition of antigen and increased density of surface pMHC^8^. The relationship between precursor frequency and affinity in GC B cells is nonlinear, but even when precursor frequencies are low, B cells can be recruited to GCs if they have sufficiently high affinity^9–11^. (3) The degree of T cell help during the GC reaction is a limiting factor on GC B cell proliferation and maturation^12,13^. Increasing the number of T follicular helper (Tfh) cells specific to epitopes on an immunogen may allow B cells into the GC that would otherwise not gain entry^14^. The modification of even a few helper T cell epitopes to relieve competition between B cell clones can have a marked impact on overall patterns of dominance^15^.

The structure of a B cell epitope as seen by the BCR likely also plays a role in immunodominance, possibly with subdominant epitopes requiring stringent or stereotyped contacts, but currently there is little experimental evidence directly addressing this topic. Computational analyses of antigen structures has focused primarily on identifying likely B cell epitopes, rather than establishing their relative immunodominance^16–18^. While immunodominance hierarchies for antigens such as HA and hepatitis C virus E2 have been experimentally mapped, the importance of epitope structure and how it might impact the trajectory of the affinity maturing B cell repertoire remains relatively undefined^1,19,20^.

In this review, we discuss various protein engineering strategies used to develop immunogens against rapidly evolving pathogens, and how they influence these three rather well-characterized elements of B cell immunodominance to preferentially elicit antibodies to subdominant epitopes.

## Consolidating protein engineering strategies into general approaches

The following sections focus on three general approaches of immunogen design. We discuss recent developments in strategies to (1) magnify the overall humoral response, (2) prevent or reduce the elicitation of ‘off-target’ antibody responses, and (3) specifically amplify responses targeting preferred epitopes. Discussion of these strategies focuses on influenza and HIV viral glycoproteins but extend to other viruses including respiratory syncytial virus (RSV), dengue, and Zika. Importantly, the strategies discussed here are not mutually exclusive, and many immunogens will likely influence immunodominance through multiple mechanisms.

## Magnification of the overall humoral response

### Multimeric display

The repetitive presentation of viral antigens, sensed by the degree of surface Ig-crosslinking, is a key factor to increasing the robustness of B cell responses^21^. Historically, multimeric display was accomplished using virus-like particles (VLPs) derived from human, insect, or plant viruses such as hepatitis B, flock house virus, and tobacco mosaic virus^22–28^. This multivalent display mimics the natural presentation of viral epitopes, and can elicit protective responses^29–32^. However, many of these display platforms are limited to presenting relatively small, linear peptides, and cannot readily present complex conformationally specific epitopes^26^.

In a key study, ferritin-based nanoparticles were engineered at the three-fold axis to display trimeric influenza HA antigens as genetic fusions. These immunogens elicited higher antigen-specific titers with increased breadth and protection relative to recombinant trimeric HAs^33^. Importantly, nanoparticle-immunized cohorts had more broadly reactive hemagglutinin inhibition (HAI), neutralization, and higher stem-directed titers, indicating that multimeric display can impact patterns of dominance in favor of cross reactive and subdominant responses^33,34^. More recently, the use of genetic fusion-based protein nanoparticle immunogens has extended beyond influenza HA; protein scaffolds displaying prefusion stabilized RSV F or HIV Env glycoproteins elicit robust neutralizing titers underscoring the utility of nanoparticle-based display as a general design strategy^35,36^. Next-generation protein-based nanoparticles involve multi-component constructs that allow for stoichiometrically precise display of multiple antigens^37–42^. Further computational and rational protein engineering led to the development of nanoparticles with tetrahedral, octahedral, and icosahedral symmetry; allowing for a greater degree of customizable antigen display based on the desired geometry^43–45^.

Additional multivalent assemblies allow for display of antigens when genetic fusions are not possible or if mixed species are required. Lipid nanoparticles or synthetic liposomes, for example, can be engineered to present multiple antigens, resulting in similar amplification of serum titers relative to recombinant protein^46,47^. Additionally, recombinantly expressed antigens can be enzymatically ‘clicked’ onto nanoparticle scaffolds (e.g., carbohydrate-based oligomers and VLPs) using the sortase transpeptidase from *Staphylococcus aureus*^48–53^. A more recent approach for conjugating antigen to nanoparticle scaffolds involves SpyTag-SpyCatcher^54^. This technology uses a split fibronectin-binding protein subdomain from *Streptococcus pyogenes* with a linear peptide tag appended on to the antigen and the remaining protein to the nanoparticle; when combined in vitro a spontaneous covalent linkage occurs via an isopeptide bond^54^. As a short peptide sequence (13 amino acids), SpyTag is readily appended to nearly any antigen of interest. This platform allows for an easily modifiable ‘plug and display’ approach, where heterologous display can be optimized to present a variety of antigens. Proof of concept studies have used diverse betacoronavirus receptor binding domains (RBDs) as well as influenza HAs^55–58^.

Despite the significant benefits of these multivalent display strategies, they are not without drawbacks. A caveat of protein-based nanoparticles and assembly domains like SpyCatcher-SpyTag is the lack of precise stoichiometric control of multiple antigens and their own innate immunogenicity. Their use introduces additional epitopes and expands the degree of interclonal competition, potentially skewing the immunodominance hierarchy away from subdominant epitopes of interest. Masking (i.e., hyperglycosylation) of such ‘scaffold’ epitopes may be necessary to limit off-target reactivity. Furthermore, multivalent antigen display on a nanoparticle may result in steric occlusion of desired epitopes due to antigen spacing and overall density; this may ultimately restrict access of potential BCRs from engaging these epitopes leading to a reduction in response despite being presented in greater number than soluble wild-type antigen^59^.

## Restricting ‘off-target’ responses

Complex protein antigens elicit diverse GCs containing B cells that recognize a range of epitopes^60,61^. In addition to magnifying the overall humoral response, immunogen design approaches are leveraged to modulate responses to target different epitopes on a given antigen. The many epitopes present on a single immunogen result in inter-clonal competition between B cells targeting both conserved epitopes of interest, and those engaging ‘off-target’ epitopes. Decreasing the size of the competing B cell pool can influence patterns of immunodominance and is accomplished in two major ways: the outright removal or steric occlusion of undesired epitopes.

### Removal of undesired epitopes

**Fig. 1:**
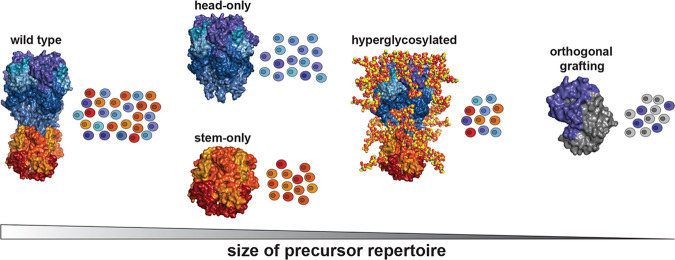
Modulating precursor repertoire with various immunogen design strategies. Schematic representation of how precursor frequency may be altered using different protein engineering strategies to remove ‘off-target’ competing clones using influenza hemagglutinin (HA) as a representative example. Colors refer to theoretical epitopes on full length HA; shades of blue and purple correspond to head-directed epitopes; shades of yellow, orange, and red correspond to stem-directed epitopes. For orthogonal grafting, a hypothetical scaffold is shown in gray with purple region illustrating a grafted HA head epitope. All images created in PyMol using PDB 5UGY.

The physical removal of undesired epitopes precludes the elicitation of responses against them, in effect modulating precursor frequency by reducing the overall number of competing B cells (Fig. 1). Many conserved epitopes recognized by B cells are conformation specific, composed of multiple segments proximal in structural space but separated in sequence space that are difficult to selectively present^62^. However, many viral proteins such as influenza HA, RSV F protein, or HIV Env can be truncated into domain-based constructs in order to minimize the inclusion of off-target epitopes while still maintaining the integrity of the target epitope^63–72^. Subsequent iterations of these constructs, such as the headless, stem-only domain ‘mini-HAs’, or the ‘engineered outer domain’ (eOD) Env constructs continue to expand the available repertoire of truncated immunogens^73,74^.

### Occlusion of off-target epitopes

Complementary to removing undesired epitopes is the steric occlusion of off-target ones, which can be done in a reversible or irreversible manner. The formation of immune complexes between antibodies and an antigen can mask epitopes; subsequent immunization with these immune complexes can sterically shield epitopes and thus bias antibody responses. This was shown for flavivirus, influenza, and HIV glycoproteins^75–78^. Recent refinement of this approach has both decreased the size of the shielding to a single chain variable fragment (scFv), or generated covalently stabilized complexes through chemical cross-linking or expression of an antigen-antibody genetic fusion^79–81^. Analogous to epitope removal, the steric shielding of off-target epitopes reduces the size of the precursor B cell pool able to bind the antigen, thus reducing the pool of potential competitors.

A commonly used method to restrict off-target responses is to introduce novel predicted N-linked glycosylation (PNG) sites on viral surface proteins to “shield” or occlude undesired epitopes. Glycans are naturally present on viral envelope proteins and play key roles in stability, pathogenicity, and immunogenicity, as well as escape from immune surveillance^82–90^. Overall glycosylation patterns can affect antigen processing, delivery into GCs, and breadth of elicited responses^91–101^. The variation in these natural glycosylation patterns in response to immune pressure provides a guide to introduce glycans on rationally designed immunogens^100,102,103^.

Recent work has underscored the effectiveness of glycan masking to restrict off-target responses across a variety of viral glycoproteins including Zika and dengue E, hepatitis C E2, influenza HA, HIV Env, and RSV F protein^36,103–107^. For flavivirus-based immunogens (e.g., Zika, dengue E), glycan shielding is critical to reduce the likelihood of antibody-dependent enhancement (ADE) mediated by cross-reactive yet non-neutralizing antibodies^104^. For influenza HA and HIV Env, the glycosylated immunogens focus responses to broadly cross reactive, yet relatively subdominant epitopes such as the HA head interface, the HA stem region, and the Env CD4bs^103,106,107^. This serum focusing is likely due to an increased proportion of the serum repertoire targeting the epitope of interest; hyperglycosylation generally does not impact overall serum titer, even for heavily glycosylated immunogens^103,107,108^. More recently, the combination of hyperglycosylation and covalent stabilization of HA trimers, where only the RBS epitope remains exposed, decreased overall serum titers without focusing to the subdominant RBS^108^. Thus, while hyperglycosylation can enrich antibody responses against desired epitopes, it is not always successful in altering immunodominance.

## Amplification of ‘on-target’ responses

In contrast to the occlusion or removal of off-target epitopes, alternative immunogen design strategies aim to expand responses towards desired epitopes. These approaches manipulate both precursor frequency and affinity, as well as epitope accessibility to preferentially target broadly conserved or protective epitopes of interest. General approaches involve the stabilization of preferred antigen conformations, glycan ‘unmasking’ of shielded epitopes, computationally inferred consensus antigens, germline targeting, and epitope resurfacing.

### Stabilization of prefusion conformation

**Fig. 2:**
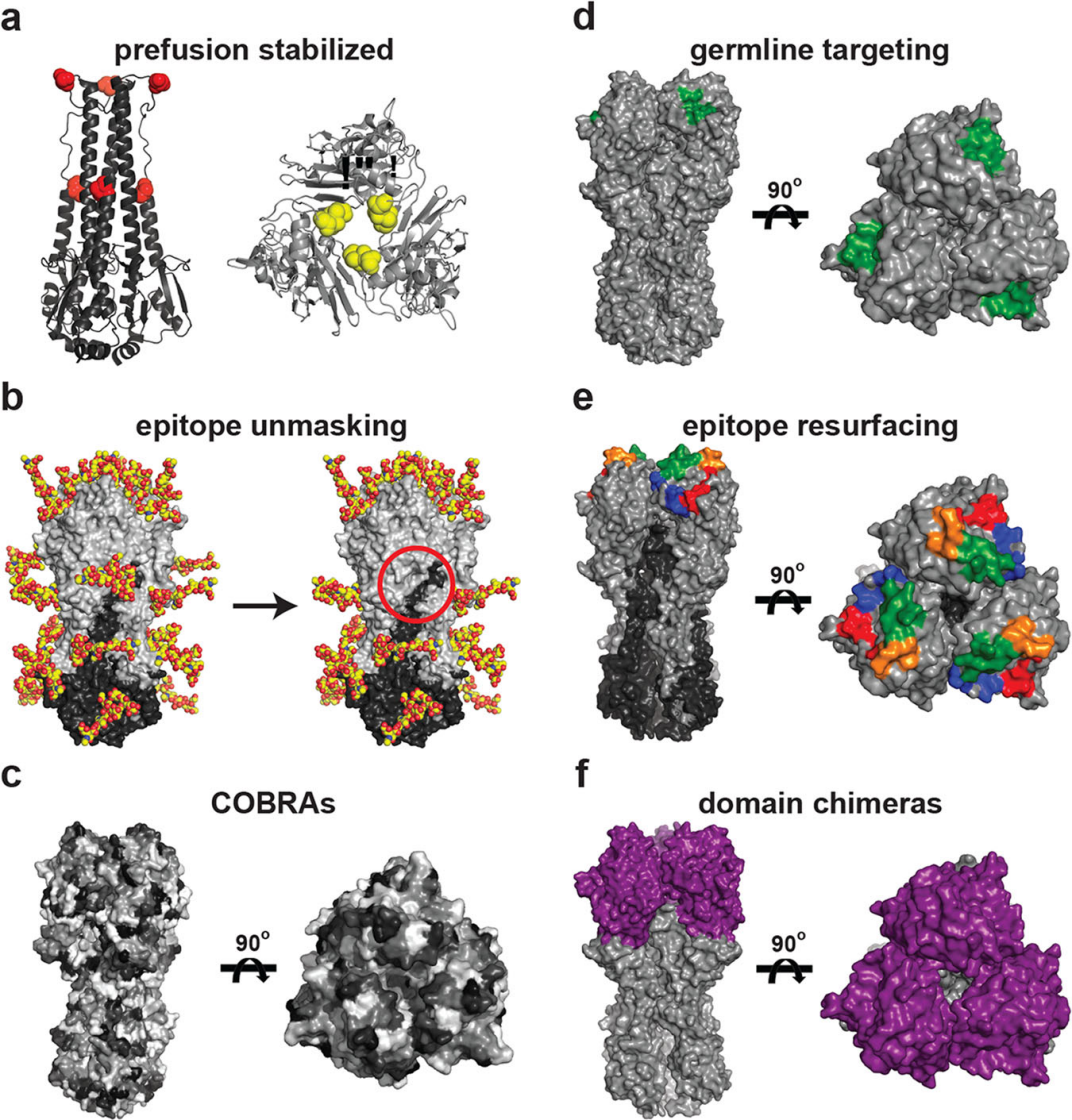
Design strategies to enhance ‘on-target’ epitope responses. **a** Stem- and head-only HAs indicating locations of engineered prolines (left, red spheres) and inter-HA cysteines (right, yellow spheres) to stabilize the prefusion conformation. **b** Selective removal of native glycans to expose neoepitopes (red circle). **c** Computational design of HA antigens based on overall subtype diversity increases cross-reactive responses. To illustrate the COBRA approach, HA is arbitrarily colored in gray shading to show variation in amino acid identity that ultimately contributes to the consensus sequence; for a complete description see Huang et al.^131^. **d** Optimization of a single epitope for a specific class of B cell precursors increases initial antigen affinity. HA receptor binding site (RBS) is shown in green as an example. **e** Resurfacing of a complex conformational-specific epitope allows for heterologous boosting of subdominant responses within memory. S1–S4 segments of the grafted RBS shown in red, orange, green, and blue, respectively. **f** Chimeric HAs where head domain (purple) of an antigenically distinct non-circulating HA is transplanted onto a conserved circulating stem domain (gray) to preferentially target stem-directed responses. All images created in PyMol using PDB 5UGY.

Viral envelope glycoproteins undergo significant conformational rearrangements between their prefusion and post-fusion states. To elicit immune responses that specifically recognize the prefusion state present on the invading virus, the glycoproteins are stabilized to prevent spontaneous structural rearrangement. This general concept was first demonstrated for influenza HA to characterize its conformational rearrangements necessary for membrane fusion either through introducing prolines or non-native cysteines to stabilize or covalently “staple” the prefusion state (Fig. 2a)^109,110^. Subsequent application of these principles for HIV Env led to the development of stabilized ‘SOSIP’ trimers through inter-subunit disulfides and conformation-locking isoleucine to proline mutations^111,112^. The initial successes with this stabilization approach has resulted in broadly applicable guidelines and even automated the design of prefusion stabilized Env antigens across viral clades^113–115^. These stabilized constructs have been instrumental in the study of HIV, and form the basis of many current Env immunogens in clinical trials^116–118^. The general strategy of prefusion stabilization has been widely implemented for other viral antigens and has informed diverse vaccine development efforts^119,120^. Specifically, the introduction of stabilizing proline residues was effective for RSV F, hMPV F, Lassa virus glycoprotein complex, ebola and Marburg glycoproteins, and several coronavirus spike proteins^121–127^. For the latter, several new iterations including ‘S-2P’ and ‘HexaPro’ in SARS-CoV-2 spike have improved efficacy and form the basis of the currently approved vaccines^127^. Such stabilization approaches help limit the overall size of the precursor pool by ensuring the prefusion conformation is dominant, and limit responses against epitopes displayed less frequently on the circulating virus.

### ‘Un-masking’ of normally occluded epitopes

The removal of naturally occurring glycans introduces new targets for antibody responses (Fig. 2b). For HIV Env and influenza HA, viral proteins with extensive surface glycosylation, selective removal of glycosylation sites around the CD4bs or HA stem epitope modulated responses to these epitopes and increased the overall breadth of elicited antibodies^128,129^. However, in some cases, glycan removal to expose neoepitopes was not sufficient to elicit broader responses, indicating that other factors influencing immunodominance are present^130^. Even if a glycan is not sterically occluding the epitope itself, it can impact the elicitation of antibodies against adjacent epitopes, as demonstrated by the increased breadth of serum responses raised against selectively de-glycosylated Env and HA antigens^106,128,129,131^.

### Computationally optimized broadly reactive antigens

While many immunogen design strategies are designed to target a single epitope, computationally optimized broadly reactive antigens (COBRAs) use a consensus sequence-based approach to optimize the entire antigen to simultaneously present multiple cross-reactive epitopes (Fig. 2c). First developed for H5 influenza HAs, COBRAs improved upon previous classes of consensus-based immunogens by removing biases from overrepresentation of certain sequence clades due to uneven sequence availability^132^. Iterative rounds of consensus sequence generation within, and then between, clades yielded HA immunogens that conferred higher HAI titers and protection against diverse H5 isolates even relative to cocktail immunizations of members from multiple H5 clades^133,134^. The COBRA approach translates to other human (e.g., H1, H2, H3), avian (e.g., H7), and swine (e.g., H1) HAs, as well as other viral antigens such as dengue virus E protein. Importantly these immunogens work in both naïve animal models and in the setting of preexisting immunity^135–147^. The exact mechanism of enhanced breadth elicited by COBRA immunogens is unknown; one possibility is increased recruitment of B cells against antigenic sites with greater cross-reactive potential leads to the enhanced diversity and breadth of responses^148,149^.

### Germline-targeting antigens

Germline biases for specific epitopes may be observed if the amino acids encoded by the naïve sequence has a threshold affinity for the antigen such that it results in a competitive advantage. For influenza HA, stem-binding antibodies are predominantly enriched for V_H_1–69, but V_H_6-1, V_H_1–18, and V_H_3–30 have also been observed; sialic acid-mimicking HA receptor binding site (RBS)-directed Abs are enriched for J_H_6 genes^150–158^. Similar gene enrichment is seen in responses to other viral glycoproteins, including Zika E (e.g., V_H_3–23/V_K_1–5), hepatitis C virus E2 (e.g., V_H_1–69), and SARS-CoV-2 spike (e.g., V_H_3–53, V_H_1–2, V_H_3–9, and V_H_3–30)^159–163^. While germline-encoded affinity can dramatically alter patterns of dominance if the precursor B cells are present with high enough frequency, this natural advantage is often insufficient in the setting of rare precursors^164^. For example, high-affinity VRC01-class precursors that target the HIV Env CD4bs are predicted to have a frequency of 1 in 0.9 million naïve B cells, and despite nanomolar affinity for rationally designed immunogens targeting these precursors, they remained minor components of the overall repertoire when present in animal models at these levels^5^.

Germline-targeting immunogens are used to preferentially bias responses towards these rare high-affinity clones (Fig. 2d). In initial studies, nanoparticles were multimerized with HIV Env eOD antigens designed to engage inferred germline precursors for VRC01-class antibodies; these immunogens activated both germline and mature VRC01 expressing cell lines^74^. Studies using VRC01 germline knock-in mice showed that elicited antibodies have VRC01-class characteristics, with varying affinity and breadth for native HIV Envs^165,166^. Subsequent generations of VRC01-class germline-targeting nanoparticles allowed for isolation and characterization of human VRC01 precursors present in naïve individuals^167^. Applying similar principles led to the development of immunogens able to engage germline-precursors for HIV Env targeting PGT121- and CH235-like broadly neutralizing antibodies, as well as those from cross-group stem-reactive influenza HA antibodies^168–170^.

Recently, germline targeting designs have been generalized to target larger pools of B cell precursors making CDRH3-mediated contacts with a given epitope^171^. This represents a conceptual shift in the approach: rather than attempting to engage the exact precursors within the repertoire, immunogens now engage a wider set of precursors that have the potential to mature breadth over the course of multiple immunizations. However, key questions remain about how to ‘guide’ BCR maturation along the desired evolutionary path, and what are the key structural characteristics of immunogens necessary for eliciting antibodies with the desired breadth^172,173^. Performing these studies in the context of adoptively transferred germline-presenting B cells, rather than a complete knock-in model, will also be necessary to understand the effects of inter-clonal competition on the ability of a rationally designed immunogen to selectively enrich for desired responses^174^.

### Epitope grafting or resurfacing

For antigenically diverse, yet structurally similar antigens, epitope ‘grafting’ or ‘resurfacing’ can be used to target subdominant responses from immunologic memory. Desired epitopes can be transplanted onto antigenically distinct scaffolds (Fig. 2e)^175^. These resurfaced constructs are thought to increase responses towards the grafted epitope due to preferential recall of memory responses against the graft versus de novo responses against the scaffold. Recently the H1 HA RBS, a complex conformationally specific epitope, was grafted onto the non-circulating avian H4 and H14 HAs. Binding to a panel of H1 RBS-directed antibodies confirmed the successful recapitulation of the grafted epitope^175^. Immunization with the resurfaced HA in mice primed with H1 HA increased the breadth of RBS-directed B cells against historical H1 isolates^175^.

A similar grafting approach was used for the HA stem region to create chimeric HAs (cHAs) whereby antigenically distinct and often non-circulating HA heads have been swapped onto stems of circulating HA subtypes (Fig. 2f)^176^. Prime-boost immunizations with these cHAs presenting head domains to which there is no preexisting immunity, but with conserved stem domains, elicited cross-reactive stem-directed antibodies capable of protecting via Fc-effector functions^177–184^. In clinical trials, cHAs show evidence of eliciting cross-reactive serum antibodies that target the HA stem in human subjects^185^. More recently, an analogous approach was used to design chimeric coronavirus spike immunogens^186^.

A complementary type of epitope resurfacing involves mutating ‘off-target’ regions to disrupt recognition of undesired epitopes. This approach has been used as a screening tool to isolate HIV Env CD4bs-directed antibodies, and flavivirus E immunogens with lower cross-reactive but non-neutralizing titers^187–189^. For example, resurfaced dengue E domain III (EDIII) immunogens preserved the broadly neutralizing 4E11 epitope while peripheral epitopes were mutated to disrupt recall responses against these regions^188,189^. Similar to masking off-target EDIII epitopes with glycans, resurfacing off-target epitopes may help prevent antibody-dependent enhancement as cross-reactive yet non-neutralizing responses are limited.

A further extension of this approach is the grafting of desired epitopes onto orthogonal protein scaffolds that lack structural homology with the wild-type antigen. In initial studies, multiple neutralizing epitopes of RSV F protein were transplanted onto orthogonal protein scaffolds. The antibody responses elicited by orthogonally-scaffolded immunogens were specific to the grafted epitopes, and had increased titers against the grafted sites despite lower titers to RSV F protein overall^190,191^. While the difficulty in designing orthogonal scaffolds significantly increases with the complexity of the target epitope, this approach completely avoids eliciting antigen-specific off-target responses.

A primary drawback of epitope resurfacing is the introduction of a significant number of neoepitopes. For example, grafting the H1 HA RBS onto an H4 full length soluble ectodomain (FLsE) HA scaffold, or grafting an antigenically distinct HA head onto a conserved HA stem domain, expands inter-clonal competition via the introduction of novel immunodominant HA head epitopes^175,176^. Likewise, while orthogonal grafting does not present any epitopes shared by that class of antigens (e.g., RSV F protein), it still presents neoepitopes which will have their own intrinsic immunodominance hierarchy. Overall, while resurfaced immunogens can elicit antibodies against desired epitopes with increased frequency or breadth, current iterations do not optimally alter patterns of dominance. The combination of epitope grafting with other design strategies is likely needed to fully realize the epitope immune-focusing potential of such constructs.

### Increasing the breadth of antibody responses

The development of next-generation vaccines is further complicated by the fact that engagement of a conserved epitope does not guarantee breadth^192^. Additional steps are therefore needed to ensure broad reactivity even if elicited antibodies engage the desired epitope. Immunogen design strategies to increase the breadth of responses can be broadly classified as single- or multi-step approaches.

### Prime and boost formulations to focus from within memory

Initial exposures to a pathogen can influence subsequent responses, a phenomenon termed “original antigenic sin”^193,194^. Inferred germline antibodies or unmutated common ancestors (UCAs) of broadly neutralizing influenza HA antibodies isolated from human donors show high affinity for strains circulating during the donor’s early childhood, suggesting that early exposures imprint responses that are subsequently matured and refined upon re-exposure^195–197^. Repeated exposure in the form of seasonal vaccination against influenza does not appear to alter the patterns of dominance established towards particular HA subtypes^175,198–200^. However, recall and maturation of HA stem-directed responses has been observed following immunization with divergent wild-type or chimeric strains, where the stem region is the conserved epitope between exposures^201–205^. Even if overall patterns of dominance are not altered, subdominant antibodies can show increased breadth upon re-exposure to antigenically similar epitopes^175^.

Computational studies suggest that there are optimal antigenic distances between primary and secondary immunogens that will help direct responses toward conserved and broadly protective epitopes^206–209^. Too little antigenic distance, and memory responses against variable epitopes will dominate the repertoire; too great a distance, and de novo responses against variable epitopes dominate. Understanding how antigenic distance impacts preferential recall of imprinted responses will be critical for designing optimized immunogens capable of expanding subdominant populations from within the memory compartment.

### Eliciting cross-reactive responses from single immunizations

Immunization with a cocktail of HA homotypic nanoparticles has been shown to elicit broader serum titers than individual nanoparticles^34^. However, the breadth of these serum responses is due to the summation of individual strain-specific responses rather than broad reactivity from specific antibodies. The simultaneous presentation of multiple antigens within a single immunogen, however, elicits broader antibodies, and in certain cases focuses antibody responses on conserved epitopes^40,41,55,56^. While the precise mechanism of how heterologous display focuses responses to cross-reactive epitopes has not yet been determined, evidence suggests that such an immunogen confers a competitive avidity advantage to cross-reactive B cells^40,41^.

**Fig. 3:**
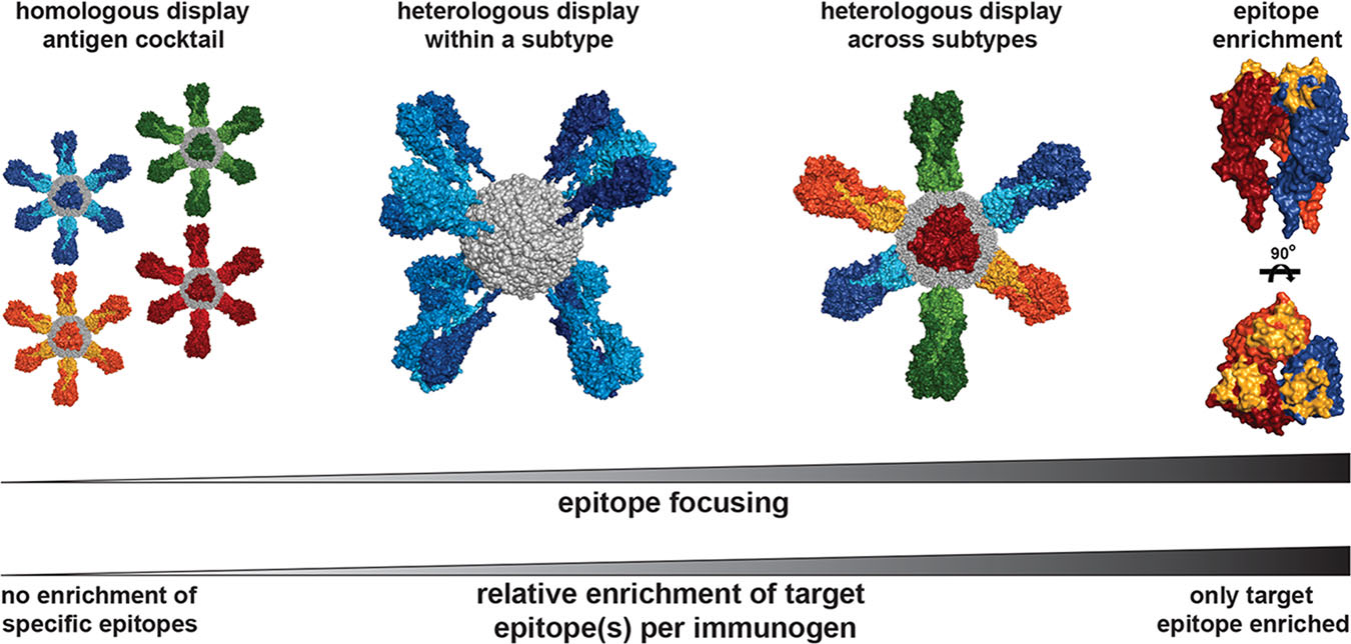
Proposed use of heterologous antigen display to focus responses toward desired epitopes. A proposed spectrum of epitope focusing immunogens incorporating various multimeric or heterologous display strategies is shown; unique colors represent antigenically distinct components. As the relative overall enrichment of the target epitope increases, so does the degree of epitope focusing. Heterologous display within a subtype presents a wider gradient of conserved epitopes due to the smaller antigenic distance between presented components. Heterologous display of antigenically distinct antigens enriches for conserved epitopes, but there are still other epitopes that are conserved between various combinations of component antigens. The endpoint of this spectrum is an epitope-enriched immunogen, where a single epitope is presented as multiple copies with all other epitopes presented once. All images created in PyMol using PDBs 5UGY and 3BVE.

However, with heterologous display, especially when presenting an array of wild-type antigens, there is often a gradient of epitope conservation that ranges from unique, to partially conserved, to fully conserved across the set of antigens being displayed. Thus, there appears to be a ‘sliding scale’ of benefit to all antigen-specific B cells that is combined with the preexisting immunodominance hierarchy to determine the overall epitope distribution (Fig. 3). If a goal is to focus antibody responses to a specific epitope, heterologous display could be optimized such that only a single epitope is present multiple times, while all other epitopes are present only once. This ‘epitope-enriched’ immunogen would, in theory, confer an avidity advantage specifically to B cells engaging the desired epitope, and thus maximize their relative competitive advantage. Heterologous display of epitope-resurfaced antigens, where antigenically distinct scaffolds present a common grafted epitope, would likely result in enhanced immune focusing.

## Future questions

Despite significant advancements in recent years, understanding immunodominance and what is required to create a vaccine with long-term efficacy against a rapidly evolving pathogen is necessary. Some key fundamental questions that remain include, but are not limited to:

1. If immunodominance hierarchies are altered by next-generation vaccines, will previously conserved subdominant epitope(s) nevertheless remain conserved in response to significant immune pressure? In other words, by focusing immune responses to a conserved epitope, will escape (i.e., mutation) occur? While many conserved regions on viral glycoproteins play important functional roles (e.g., receptor binding, membrane fusion), key residues are often a fraction of the entire eptiope^210–212^.
2. Can B cell populations targeting a particular conserved epitope be guided towards breadth through serial immunizations with rationally designed constructs, or is the potential for breadth intrinsic to a specific subset of clones? This has significant implications for pathogens such as HIV, where many bnAbs have structural features (e.g., CDR length) that greatly restrict the size of precursor populations.
3. Are there structural, epitope-specific factors that influence immunodominance hierarchies? The major components of precursor frequency and affinity, T cell help, and epitope accessibility do not account for the impact of the epitope itself as a substrate during affinity maturation. For example, an HA receptor-mimicking RBS-directed antibody makes contacts within the sialic acid binding pocket, a recessed epitope surrounded by a highly variable periphery, where acquired mutations in CDRs can affect or completely abrogate binding^154^. In contrast, an HA lateral patch-binding antibody makes contacts with a mostly continuous hydrophilic surface, where the similar CDR mutations are less likely to disrupt binding^213^. In effect, the affinity maturing antibodies may follow evolutionary paths, determined by the targeted epitope. Over time, the relative frequency of beneficial, neutral, or deleterious mutations impact the size of competing clonal B cell pools, and thus the trajectory of inter-clonal competition.

## Conclusion

Current protein engineering strategies, like those described above, allow tailoring of an immunogen to elicit the desired antibody response. Importantly, many of the described approaches take orthogonal and therefore complementary approaches to immune-focusing. For example, a germline-targeting immunogen, with hyperglycosylated periphery, displayed on a nanoparticle leverages three different strategies: (1) the multimeric display, (2) steric shielding of ‘off-target’ and nanoparticle scaffold epitopes, and (3) epitope optimization to engage a set of precursor B cells. Multimeric display increases the overall size of the precursor B cell pool and the avidity of antigen interactions, steric shielding of undesired responses leads to the reduction of inter-clonal competition from undesired clones, and germline targeting ensures a select set of precursors have increased affinity and thus a competitive edge in the GC reaction. While the intrinsic immunodominance hierarchies for specific antigens will likely determine the optimal approach needed to expand broadly protective responses, the above strategies allow for selective manipulation of the major contributing factors to immunodominance.

These protein engineering approaches can be similarly used to generate probes to deconvolute the GC reaction. Selective resurfacing or occlusion of epitopes, the modulation of endogenous/exogenous T cell epitopes, display on oligomeric scaffolds, and other rational design strategies, provide an opportunity to perform mechanistic experiments investigating underlying B-cell biology. Simultaneous basic and translational advancements will allow for iterative improvement of pre-clinical vaccine candidates in the pursuit of next-generation viral vaccines for rapidly evolving pathogens.
